# De-escalation of breast surgery for early stage breast cancer

**DOI:** 10.1093/jjco/hyag098

**Published:** 2026-07-01

**Authors:** Takeshi Murata, Tomomi Fujisawa, Fumikata Hara, Toshiyuki Ishiba, Yukinori Ozaki, Takehiko Sakai, Yasuaki Sagara, Hideo Shigematsu, Akihiko Shimomura, Kazuki Sudo, Kaori Terata, Yoichi Naito, Kazuki Nozawa, Tadahiko Shien

**Affiliations:** Department of Breast Surgery, National Cancer Center Hospital, 5-1-1 Tsukiji, Chuo-ku, Tokyo 104-0045, Japan; Breast Oncology, Gunma Prefectural Cancer Center, 617-1 Takabayashi-Nishimachi, Ota-shi, Gunma 373-8550, Japan; Breast Oncology, Aichi Cancer Center, 1-1 Kanokoden, Chikusa-ku, Nagoya-shi, Aichi 464-8681, Japan; Department of Breast Surgery, Institute of Science Tokyo Hospital, 1-5-45 Yushima, Bunkyo-ku, Tokyo 113-8519, Japan; Department of Breast Medical Oncology, Breast Oncology Center, Cancer Institute Hospital, Japanese Foundation for Cancer Research, 3-8-1, Ariake, Koto-ku, Tokyo 135-8550, Japan; Department of Breast Surgical Oncology, Cancer Institute Hospital of Japanese Foundation for Cancer Research, 3-8-1, Ariake, Koto-ku, Tokyo 135-8550, Japan; Department of Breast and Thyroid Surgical Oncology, Sagara Hospital, 3-31 Matsubara-cho, Kagoshima-shi, Kagoshima 892-0833, Japan; Department of Surgical Oncology, Research Institute for Radiation Biology and Medicine, Hiroshima University, 1-2-3 Kasumi, Minami-ku, Hiroshima-shi, Hiroshima 734-8551, Japan; Department of Breast and Medical Oncology, National Center for Global Health and Medicine, 1-21-1 Toyama, Shinjuku-ku, Tokyo 162-8655, Japan; Department of Medical Oncology, Department of International Clinical Development, National Cancer Center Hospital, 5-1-1 Tsukiji, Chuo-ku, Tokyo 104-0045, Japan; Department of Breast and Endocrine Surgery, Akita University Hospital, 44-2 Hasunuma, Hirosawa, Akita-shi, Akita 010-8543, Japan; Department of General Internal Medicine, National Cancer Center Hospital East, 6-5-1 Kashiwanoha, Kashiwa-shi, Chiba 277-8577, Japan; Department of Advanced Clinical Research and Development, Nagoya City University Graduate School of Medical Sciences, 1 Kawasumi, Mizuho-cho, Nagoya-shi, Aichi 467-8602, Japan; Department of Breast and Endocrine Surgery, Okayama University Hospital, 2-5-1 Shikata-cho, Kita-ku, Okayama-shi, Okayama 700-8558, Japan

**Keywords:** breast cancer, de-escalation, non-surgical ablation, active surveillance, review

## Abstract

Breast cancer treatment is rapidly evolving toward precision-based de-escalation strategies for oncological safety and minimizing treatment-related morbidity. In early stage disease, minimally invasive approaches, such as non-surgical ablation techniques, including cryoablation and radiofrequency ablation, potentially constitute alternatives to conventional surgery for carefully selected low-risk tumors. Active surveillance trials challenge the necessity of immediate surgery for low-risk ductal carcinoma in situ, reflecting a paradigm shift toward risk-adapted management. In the neoadjuvant setting, achieving radiologic and pathologic complete responses, particularly in HER2-positive and triple-negative subtypes, is being investigated to determine the feasibility of omitting breast surgery under strict imaging and biopsy-guided protocols. Circulating tumor DNA-based minimal residual disease assays offer a promising tool for refining risk stratification and potentially guiding the extent of surgery and adjuvant therapy. These strategies represent a transformative movement toward biologically tailored, response-adapted, less invasive treatments for early breast cancer to optimize quality of life without compromising long-term outcomes.

## Teaser

Precision-based de-escalation strategies in early breast cancer—including ablation, active surveillance, response-guided surgery omission, and ctDNA-based risk stratification—aim to reduce treatment invasiveness while maintaining oncologic safety.

## Introduction

The standard treatment for breast cancer combines local therapy, surgery, and radiation therapy with systemic therapy; the goal of surgery is to achieve local control in the breast and axilla. Clinical trials have shown that extended mastectomy does not improve prognosis [1]. Subsequent studies have demonstrated no difference in survival between total mastectomy and breast-conserving surgery [2–7], thereby providing viable surgical options for breast preservation. Moreover, non-surgical ablation techniques such as radiofrequency ablation (RFA) and cryoablation have emerged for certain early stage breast cancers. In Japan, RFA received regulatory approval in July 2023 based on the results of the multicenter collaborative Radiofrequency Ablation Therapy for Early Breast Cancer as Local Therapy (RAFAELO; jRCTs032180229) trial [8], which evaluated the efficacy and standardization of RFA for early breast cancer; RFA has been included under insurance coverage since December 2023, as discussed further. Regarding axillary surgery, the introduction of sentinel lymph node biopsy (SLNB) demonstrated, in a clinical trial (NSABP-B32 [9]), that axillary lymph node dissection (ALND) does not improve prognosis in cN0 patients with sentinel node-negative breast cancer. This established SLNB as the standard treatment for cN0 breast cancer. Even in cases with limited sentinel node metastases, additional ALND did not improve prognosis (ACOSOG Z0011 [10], AMAROS [11], IBCSG 23–01 [12], OTOASOR [13], SENOMAC [14] trials); thus, in clinical settings, ALND is omitted in patients with minimal sentinel node involvement. Neoadjuvant chemotherapy (NAC) remains the standard treatment for patients requiring perioperative systemic therapy. Advances in systemic therapy have increased the number of patients achieving a pathological complete response (pCR) in the breast and axilla. For patients who do not achieve pCR, a residual disease-guided approach wherein adjuvant therapy is selected based on disease pathology improved prognosis in multiple clinical trials (CREATE-X [15], KATHERINE [16], and OlympiA [17] trials). Consequently, NAC-based breast cancer treatment has become increasingly common among eligible patients. Given this background, various strategies for the de-escalation of breast and axillary surgeries are being investigated. These include non-surgical ablation as a minimally invasive surgery for cN0 early breast cancer, surgery omission in low-risk ductal carcinoma in situ (DCIS), SLNB omission in cN0 breast cancer treated with upfront surgery, tailored axillary surgery (TAS) for axillary de-escalation during upfront surgery for clinically node-positive breast cancer, omission of breast surgery in patients achieving a clinical complete response (cCR) after NAC, and post-NAC axillary surgery de-escalation. This review discusses the de-escalation of breast surgeries in detail.

## Non-surgical ablation as minimally invasive surgery for cN0 early breast cancer

Non-surgical ablation for cN0 early breast cancer is an innovative, minimally invasive treatment approach that is aimed at eliminating early stage breast cancer without the need for traditional surgical excision. Treatments, such as RFA and cryoablation, have received attention for their potential to provide safer and less invasive alternatives, particularly for those with cN0 early breast cancer.

The RAFAELO study is a prospective, multicenter, single-arm, confirmatory trial that was designed to evaluate the safety and efficacy of breast RFA in women aged 20–79 years with small, unifocal breast cancers (≤1.5 cm). Eligible patients had invasive ductal carcinoma (IDC) or DCIS diagnosed by core needle biopsy, regardless of tumor grade, hormone receptor (HR) status, or HER2 status. The primary endpoint was ipsilateral breast tumor recurrence (IBTR)-free survival at 5 years. A total of 372 patients were enrolled, 370 of whom underwent successful RFA. After a median 5-year follow-up, IBTR was observed in 2 patients, yielding a 5-year IBTR-free survival rate of 98.6% (90% CI: 97.1%–99.3%). All primary endpoints were met. Adverse events included grade 1 burns in 4 patients (1.1%) and grade 2 burns in 3 patients (0.8%). No severe adverse events were reported. The study suggests that RFA is a promising, minimally invasive alternative to surgery with potential advantages in cosmetic outcomes and patient satisfaction [8].

The Cryoablation of Low-Risk Breast Cancer Less Than 1.5 Cm: An Evaluation of Local Recurrence (ICE3; NCT02200705) trial is a prospective, multicenter, non-randomized trial that was conducted to evaluate the safety and efficacy of cryoablation as a non-surgical treatment for low-risk, early stage breast cancer in women aged ≥60 years. The trial included women with unifocal, ultrasound-visible IDC ≤1.5 cm, HR-positive, HER2-negative, and histologic grade 1–2. The primary endpoint was the 5-year IBTR rate. In total, 194 patients underwent successful cryoablation therapy. The mean age was 74.9 years, and the mean tumor size was 7.4 mm. At 5 years, the IBTR and breast cancer survival rates were 4.3% and 96.7%, respectively. Among the patients who received endocrine therapy, the IBTR rate was 3.7%. The minor (88.2%) and moderate (9.6%) adverse events were mild and resolved without any residual effects. A significant improvement in distress was observed at 6 months (*p* < 0.001). Thus, cryoablation is a promising non-surgical alternative for low-risk patients; however, further studies are needed to confirm its viability [18] (Table 1).

**Table 1:** Clinical trials of non-surgical ablation.

**Trial**	**Country/Region**	**Design**	**Intervention**	**Key eligibility**	**Primary endpoint**	**No. of patients**	**Current status/Key findings**
RAFAELO [8] (jRCTs032180229)	Japan	Prospective, Multicenter, Single-arm	Radiofrequency ablation (RFA)	Women aged 20–79 with unifocal, MRI and ultrasound-visible invasive ductal carcinoma (IDC) or ductal carcinoma in situ (DCIS) ≤1.5 cm, diagnosed by preoperative core-needle biopsy	IBTR-free survival at 5 years	372 patients enrolled	5-year IBTR-free survival: 98.6% (90% CI 97.1%–99.3%). Two patients experienced IBTR. Device-related adverse events: 1.1% with Grade 1 burns, 0.8% with Grade 2 burns.
ICE3 [18] (NCT02200705)	United States	Prospective, Non-randomized, Multicenter	Cryoablation	Women ≥60 years with unifocal, ultrasound-visible invasive ductal carcinoma ≤1.5 cm, grade 1–2, HR-positive, HER2-negative	IBTR-free survival at 5 years	194 patients treated	5-year IBTR-free survival: 95.7%, with breast cancer survival rate of 96.7%. No serious device-related adverse events were reported. Minor (88.2%) and moderate (9.6%) adverse events resolved without residual effects. Quality of life improved significantly (*P* < .001).

### Surgery omission in low-risk DCIS

With the widespread implementation of mammographic screening, DCIS accounts for 20%–30% of newly diagnosed breast cancer cases worldwide [19]. The standard management of DCIS consists of breast surgery, with or without axillary staging, followed by adjuvant therapy when indicated. However, concerns have been raised regarding the potential overtreatment of patients with biologically low-risk DCIS. Consequently, several prospective clinical trials, namely the Low-Risk DCIS (LORIS) [20], LOw Risk DCIS (LORD; NCT02492607) [21], Comparison of Operative versus Monitoring and Endocrine Therapy (COMET; NCT02926911) [22], and Low-risk DCIS With Endocrine Therapy Alone-TAM (LORETTA; JCOG1505) [23, 24], are currently investigating the safety and feasibility of omitting surgery in selected patients with low-risk DCIS.

The COMET trial is a prospective, randomized, pragmatic clinical trial that was conducted in the United States to evaluate the safety of active surveillance for patients with low-risk DCIS [22]. Eligible patients had low- or intermediate-grade DCIS diagnosed using core- or vacuum-assisted biopsy (VAB) without evidence of invasive cancer. Estrogen receptor (ER) positivity was not mandatory, and limited comedo necrosis was allowed; thus, the COMET trial is the most inclusive and real-world-oriented DCIS de-escalation trial. The participants were randomized to active surveillance (clinical and imaging follow-ups; optional endocrine therapy) or guideline-concordant care (surgery, radiotherapy, and endocrine therapy). The primary endpoint was the incidence of ipsilateral invasive breast cancer. At early follow-up, no statistically significant difference was observed in the rate of ipsilateral invasive breast cancer between the active surveillance and guideline-concordant care groups. OS and breast cancer-specific survival were excellent and comparable in both groups. Patients in the active surveillance arm reported better quality of life and less treatment-related morbidity. A substantial proportion of the patients in the surveillance arm avoided surgery during the early follow-up period. Long-term follow-up is ongoing to confirm the reliability of these findings.

The LORETTA trial is a prospective clinical study that was conducted in Japan by the Japan Clinical Oncology Group [23, 24]. This study evaluated the feasibility of omitting surgery in patients with low-risk DCIS. Eligible patients had ER-positive DCIS with a low-to-intermediate nuclear grade and no comedo necrosis. Participants were treated with endocrine therapy alone, without surgery or radiotherapy. The primary endpoint was the incidence of ipsilateral invasive breast cancer. The secondary endpoints were overall survival (OS) and safety. At the 5-year follow-up, the cumulative incidence of invasive breast cancer was ~10%. No breast cancer-related deaths were reported during the study period. However, the trial was terminated early because the observed invasive cancer rate did not meet the predefined criteria for continuation. This early termination highlighted concerns regarding oncologic safety despite favorable survival outcomes. The results suggest that surgery omission may be feasible in highly selected patients but carries a measurable risk of invasive progression [24]. Overall, LORETTA highlights the need for careful patient selection and randomized trials before changing the standard DCIS management.

The LORIS trial was a randomized controlled study conducted in the United Kingdom that evaluated active surveillance versus standard surgery for low-risk DCIS [20]. Eligible patients had screen-detected, low-, or intermediate-grade DCIS that was diagnosed using core biopsy. The participants were randomized to either surgery or close observation with regular imaging and clinical follow-up. The primary endpoint was the development of ipsilateral invasive breast cancer. To date, the main trial results have not been fully published, and long-term outcomes are awaited.

The LORD trial is a multicenter, randomized, non-inferiority study conducted across several European countries [21]. It focused on patients with low-grade ER-positive DCIS detected by screening. Patients were assigned to receive either standard surgical treatment or active surveillance without immediate surgery. The primary endpoint was the incidence of ipsilateral invasive breast cancer during the long-term follow-up. The trial’s results are awaited, and the study is ongoing or awaiting mature outcome data (Table 2).

**Table 2:** Clinical trials of surgery omission in low-risk DCIS.

**Trial**	**Region**	**Design**	**Intervention**	**Key eligibility**	**Primary endpoint**	**No. of patients**	**Current status/Key findings**
LORIS [20]	United Kingdom	Randomized controlled trial	Active surveillance vs standard surgery	Screen-detected DCIS, low/intermediate nuclear grade, no comedo necrosis, no mass lesion	Ipsilateral invasive breast cancer incidence	188	Accrual completed early; long-term follow-up ongoing
LORD [21] (NCT02492607)	The Netherlands/Europe	Randomized controlled trial/Patient-preference trial	Active surveillance vs standard surgery ± RT ± ET	Low-grade DCIS, age ≥ 45, screen-detected, ER-positive, no high-grade features	Ipsilateral invasive breast cancer incidence	~1240 patients (~620 per arm)	Ongoing; modified to include patient-preference design
COMET [22] (NCT02926911)	United States	Randomized controlled trial	Active monitoring vs guideline-concordant care	Low-risk DCIS (grade 1–2, ER+), no mass, screen-detected	2-year cumulative incidence of ipsilateral invasive breast cancer	Active monitoring: 497 Guideline-concordant care: 498	Accrual completed; Non-inferiority demonstrated at 2 years; QoL benefit observed
LORETTA [23, 24] (JCOG1505)	Japan	Single-arm confirmatory trial	Endocrine therapy alone (tamoxifen), no surgery	ER-positive, low-risk DCIS, low/intermediate grade, no comedo necrosis	5-year cumulative incidence of ipsilateral invasive breast cancer	341	Accrual completed; low invasive cancer rate (9.8%), no mortality reported

The COMET and LORETTA trials shared several common challenges in the non-surgical management of low-risk DCIS. Accurate risk stratification is a key issue because the current clinical and pathological criteria may not reliably identify patients with a negligible risk of progression to invasive disease. Furthermore, both trials faced concerns regarding the cumulative invasive cancer incidence with longer follow-up despite favorable short-term outcomes. In addition, patient adherence to active surveillance and the psychological burden of living with unresected cancer remain important considerations. Future perspectives include refinement of patient selection using molecular biomarkers and imaging advances, as well as long-term outcome data to determine whether surgery omission can be safely incorporated into standard DCIS management.

### Omission of breast surgery in patients with post-NAC cCR

A prospective multicenter phase II study evaluated whether breast surgery could be omitted in selected patients with early breast cancer after NAC [25, 26]. Patients had unicentric cT1–2, N0–1, M0 HER2-positive, or triple-negative disease and an excellent imaging response after NAC. Image-guided VAB was performed. If no residual invasive or in situ disease was found, breast surgery was omitted, and whole-breast radiotherapy was delivered. Among 50 enrolled patients with HER2-positive or triple-negative breast cancer, 31 (62%; 95% CI 47.2%–75.3%) had VAB-confirmed pCR. At a median follow-up of 55.4 months, the IBTR rate was 0%, and the 5-year disease-free survival (DFS) and OS rates were 100% in patients without surgery. These results suggest that surgery omission may be feasible in carefully selected patients with biopsy-confirmed pCR [25, 26].

JCOG1806 (jRCTs031190129) is an ongoing single-arm trial conducted by the Japan Clinical Oncology Group to evaluate non-surgical management in patients with HER2-positive early breast cancer (cT1–2N0M0) who achieve a cCR after primary systemic therapy (PST). PST is a term used in several clinical trials and corresponds to NAC in the context of this review. In an exploratory analysis of 340 registered patients, 196 (57.6%) achieved cCR, as assessed by physical examination and imaging (biopsy was mandatory for HR-positive breast cancer). cCR was significantly less common in patients with ER-positive tumors. This analysis focused on predictive factors and cCR rates, not definitive surgery omission outcomes; however, long-term survival endpoints are awaited [27].

The OPTIMIST trial is a prospective, multicenter, single-arm, non-inferiority study designed to assess whether breast surgery can be safely omitted in patients predicted to have pCR based on MRI and VAB after neoadjuvant systemic therapy (NST). NST is a term commonly used in clinical trial settings and is equivalent to NAC within the framework of this review. The trial is set to enroll 533 patients, assuming a 5-year DFS of ~88% as a historical control with a non-inferiority margin of 4% and 80% power. Eligibility uses post-NAC MRI criteria (residual ≤1.0 cm and lesion-to-background signal ≤1.6) and VAB confirmation; breast surgery is omitted if no residual tumor is found. The primary endpoint is 5-year DFS, with an interim analysis planned at 50% enrolment. The actual outcome data have not yet matured/been published [28] (Table 3).

**Table 3:** Clinical trials of omission of breast surgery in patients with post-NAC cCR.

**Trial**	**Country/Region**	**Design**	**Intervention**	**Key eligibility**	**Primary endpoint**	**No. of patients**	**Current status/Key findings**
MDACC Selective Elimination of Breast Surgery [25, 26]	United States (MD Anderson Cancer Center)	Phase II, prospective, single-arm	Omit breast surgery → whole-breast RT ± boost. Axillary surgery omitted in initially cN0; limited surgery in cN+.	cT1–2 N0–1 M0; HER2+ or TNBC; excellent radiologic response after NAC; pCR confirmed by image-guided vacuum-assisted biopsy (VAB)	Ipsilateral breast tumor recurrence	~50 enrolled 31 surgery-omission cohort	At median 55.4 months follow-up, 0% ipsilateral breast recurrence, 100% DFS/OS in surgery-omission cohort; demonstrates feasibility in highly selected patients.
JCOG1806 [27] (AMATERAS-BC)	JAPAN	Phase III, single-arm confirmatory study	No breast and axillary surgery → whole-breast RT + boost + continuation of anti-HER2 therapy	cT1–2N0M0 HER2+ IDC; clinical complete response (cCR) by imaging and exam after NAC	3-year distant metastasis-free survival (DMFS)	353 patients enrolled	Accrual completed; follow-up ongoing. The overall cCR rate: 57.6% (95% CI 52.2%–63.0%). Twenty-eight patients with a non-cCR had no residual invasive or in situ carcinoma in the breast upon pathological examination
OPTIMIST [28] (NCT05505357)	South Korea	Prospective, multicenter, single-arm non-inferiority	Omit breast surgery, RT mandatory Axillary surgery may be omitted in cN0 or ycN0 patients	HER2+, TNBC, or ER-low; clip placed pre-NAC; predicted pCR by MRI criteria; no residual tumor on VAB	5-year disease-free survival (DFS)	533 planned	Recruiting

The confirmation of pCR post-NAC is essential for determining the treatment strategy for patients, particularly regarding whether surgery can be omitted. The NRG-BR005 trial incorporated advanced biopsy techniques, including VAB, in addition to conventional methods such as fine-needle aspiration (FNA) and MRI for evaluation [29]. This trial demonstrated that VAB was particularly effective in identifying residual disease and accurately confirming pCR. The accurate assessment of residual disease via biopsy is critical for making informed decisions regarding surgical omission.

### Potential molecular response-guided surgery

Circulating tumor DNA (ctDNA)-based minimal residual disease (MRD) assays are being actively studied for various cancer types [30] and are expected to play an increasingly important role in breast cancer diagnosis and treatment strategies [31]. In recent years, patterns of ctDNA clearance, persistence, and reappearance (ctDNA dynamics) before, during, and after NAC have been shown to correlate with post-NAC pCR and prognosis [32, 33]. Particularly in triple-negative breast cancer, a strong correlation has been observed between ctDNA negativity during and at the end of NAC with the pCR rate [34, 35]. Moreover, ctDNA negativity at the end of NAC is a stronger prognostic factor than pCR and is associated with lower rates of distant recurrence [35].

Currently, decisions regarding the reduction of breast and axillary surgeries in ongoing clinical trials are based on cCR assessment using imaging, including MRI, after NAC, as well as confirmation of the absence of local residual disease through VAB/core biopsy. However, discrepancies between imaging and pathology, as well as sampling errors in needle biopsies, remain significant challenges. By incorporating ctDNA analysis before and after NAC, the possibility of sampling errors can be minimized, and there is an additional advantage in assessing MRD both locally and systemically.

Further studies that incorporate ctDNA-based evaluation alongside imaging and tissue biopsy for post-NAC cCR assessment could potentially allow safer, more accurate selection of cases for reduction or omission of breast and axillary surgeries. To validate this hypothesis, early planning and implementation of randomized controlled trials are necessary.

## Limitations

### Selection bias, follow-up duration, and generalizability

Some limitations of the current evidence should be acknowledged. Many de-escalation studies are single-arm or non-randomized in design, which introduces inherent selection bias and limits the ability to draw definitive causal inferences. In addition, follow-up durations in most contemporary trials remain relatively short, making it difficult to fully assess long-term local control and survival outcomes. Furthermore, many studies enroll highly selected patient populations with favorable tumor biology or excellent treatment responses; therefore, the generalizability of these findings to broader real-world populations should be interpreted with caution.

### Potential false-negative risk of image-guided biopsy after NAC

When considering omission of breast surgery after NAC, the potential false-negative risk of image-guided biopsy must be carefully considered. Even in cases with radiologic complete response, image-guided core needle biopsy may fail to detect small foci of residual disease, and reported false-negative rates vary across studies. Microscopic residual disease may therefore remain undetected despite negative biopsy results, highlighting the need for rigorous patient selection and standardized biopsy protocols before surgical omission can be safely implemented. This limitation is particularly relevant in ongoing de-escalation trials that rely on image-guided biopsy to determine surgical eligibility. Notably, the JCOG1806 trial is primarily based on cCR assessment using contrast-enhanced breast MRI and ultrasonography, with needle biopsy performed in selected cases when clinically indicated. Given that radiologic cCR does not fully exclude the presence of small residual disease, the study design explicitly accounts for this possibility. To address this concern, patients receive whole-breast radiotherapy including boost irradiation to the tumor bed, with the expectation that radiotherapy may provide adequate local control even in the presence of minimal residual foci. Accordingly, the rate of post-radiotherapy local recurrence is included as a key secondary endpoint, and the trial results will be important in determining whether this strategy can effectively compensate for the potential limitations of response assessment after neoadjuvant therapy.

### Long-term oncologic safety

Although several ongoing trials have reported encouraging short-term outcomes, long-term oncologic safety remains insufficiently established. Data regarding durable local control, distant recurrence, and OS remain immature, and extended follow-up will be essential to confirm whether de-escalation strategies can maintain oncologic outcomes comparable to those of standard surgical management. Until such evidence becomes available, interpretation of early results should remain appropriately cautious. This is especially important because many current trials have not yet reached their planned long-term endpoints.

### Practical challenges for ctDNA implementation: cost, standardization, and accessibility

Although ctDNA-guided approaches hold considerable promise for individualized de-escalation strategies, several practical challenges must be addressed before widespread clinical implementation. These include the high cost of testing, lack of assay standardization across platforms, and variability in accessibility and reimbursement among institutions and regions. Overcoming these barriers will be critical to ensuring equitable and reliable integration of ctDNA into future clinical decision-making.

In conclusion, the emerging strategies for de-escalation in early breast cancer treatment reflect a shift toward more personalized and less invasive approaches. By selectively omitting or minimizing surgery, patient morbidity can be reduced while maintaining oncological safety. These advances, including non-surgical ablation, TAS, and omission of surgery in low-risk cases or after NAC, offer promising potential for improving the quality of life of patients. Further clinical trials with long-term follow-up are necessary to confirm the safety and efficacy of these approaches. Ultimately, these evolving practices highlight the need for precise cancer treatment to achieve optimal outcomes with minimal intervention.
